# A case report of a brain abscess due to *prevotella oris* and a review of the literature

**DOI:** 10.1186/s12879-023-08306-9

**Published:** 2023-09-27

**Authors:** Chenlu Li, Chaonan Lv, Miao Yu, Yajing Zhang, Ling Ling, Wei Yue

**Affiliations:** 1 Clinical College of Neurology, Neurosurgery and Neurorehabilitation, Tianjin Medical University, Tianjin, China; 2 Department of Neurology, Tianjin Huanhu Hosipital, 6 Jizhao Rd, Tianjin, 300350 China

**Keywords:** Brain abscess, *Prevotella oris*, Nasosinusitis, Metronidazole therapy

## Abstract

**Background:**

Brain abscesses caused by *Prevotella oris* are rarely reported. Here, we described a case of a brain infection caused by *Prevotella oris* that was detected by metagenomic next-generation sequencing (mNGS).

**Case presentation:**

A 63-year-old man with no medical history reported headache in the right frontotemporal region, fever, and intermittent diplopia. Magnetic resonance imaging (MRI) revealed abnormal signals and enhancement changes in the superior sellar region. mNGS testing showed that cerebrospinal fluid collected from the spine was positive for *Prevotella oris*. After receiving a combined treatment of antibiotic therapy, the patient recovered well.

**Conclusion:**

We reviewed the relevant literature and summarized the characteristics and prognosis of this type of bacterial infection to provide ideas for clinicians to diagnose and treat this disease.

## Introduction

A brain abscess is a focal suppurative process in the brain parenchyma and remains a significant health care problem in developing countries. Pathogenic studies have shown that aerobic bacteria such as streptococci and staphylococci predominate among the confirmed pathogens of brain abscesses. A brain abscess can be caused by anaerobic bacteria that spread from parameningeal foci of infection in paranasal, odontogenic, or middle ear localizations and are often overlooked because their isolation is more difficult and requires special methods [1]. The most common anaerobic bacteria include *Cutibacterium. acnes*, *Parvimonas micra*, *Prevotella* species and *Fusobacterium* species [2]. We report an unusual case of a brain abscess caused by *Prevotella oris* with nasosinusitis. Included with this case report is a literature review of cases consistent with *Prevotella oris*.

## Case Report

A 63-year-old male patient presented with pain in the right frontotemporal region accompanied by fever with a maximum temperature of 39 °C 15 days prior to admission; he had intermittent diplopia shortly after admission; and, 5 days prior to admission, he had showed unresponsiveness. There was no previous history of infections, surgery or trauma.

**Fig. 1:**
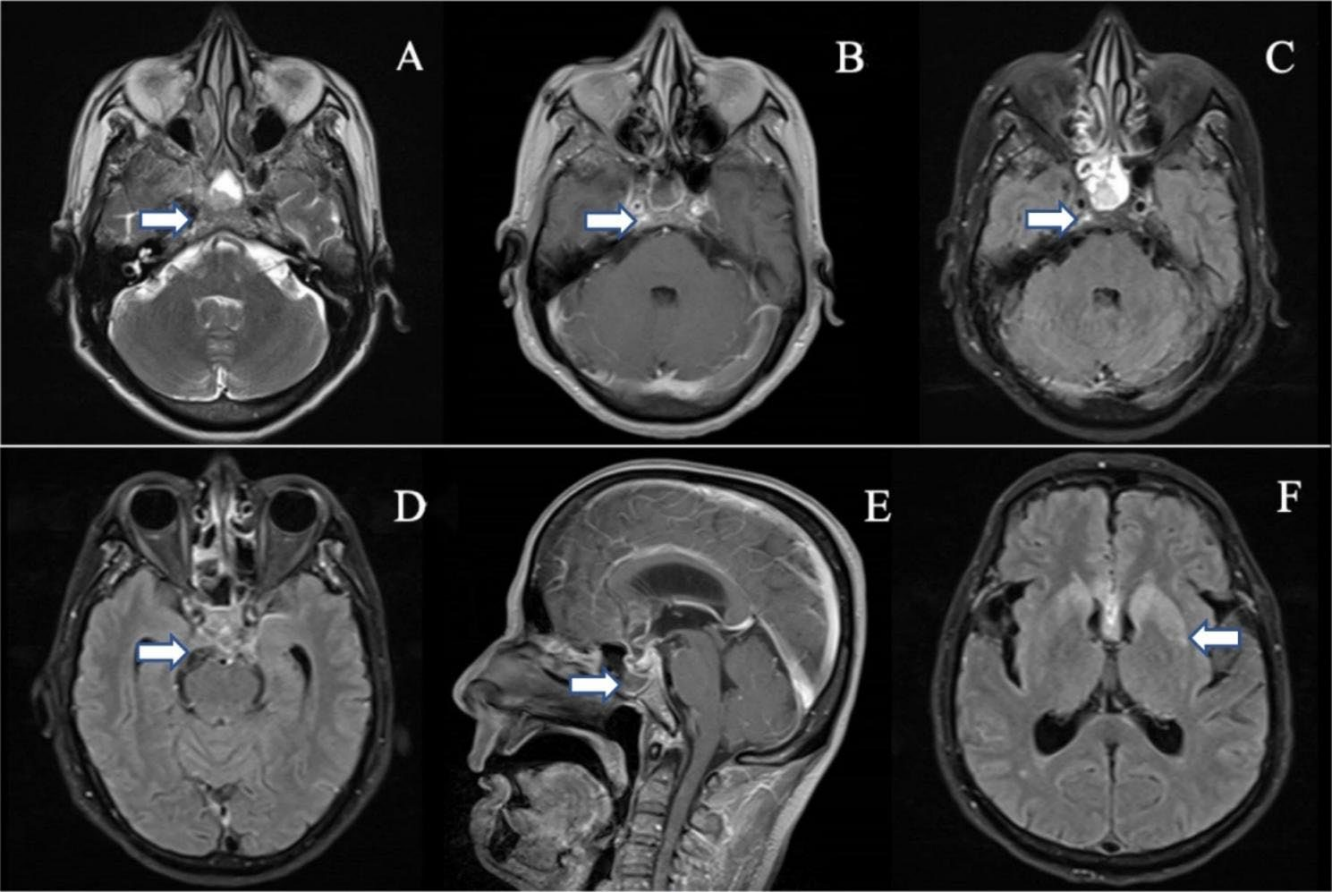
MRI of the brain: A: Slice of irregular and slightly long T2 signal in the sellar area. B, C: Slice of irregular enhancement changes in the sellar region. D: High signal in the flair sequence in the saddle region. E: Slice of irregular enhancement changes in the sellar and suprasellar regions. F: High signal in the flair sequence in the bilateral basal ganglia regions

At the time of consultation, his vital signs were stable, and his cardiopulmonary and abdominal investigations were normal. The neurologic examination showed a somnolent state, with the patient answering off the point, and he had dysarthria. The cranial nerve examination was normal, but neck rigidity, weakened muscles of the limbs and positive Kernig’s sign on the left side were noted. A brain MRI was performed and revealed abnormal signals in the bilateral basal ganglia, bilateral periventricular and corpus callosum; abnormal signals and enhancement changes in the sellar region, superior sellar region and left medial temporal region; abnormal signal enhancement in the anterior longitudinal fissure and ventral brainstem; and inflammatory signals in the right ethmoid and sphenoid sinuses (Fig. 1A-F). Routine laboratory studies showed mostly normal findings, with the exception of slightly elevated D-dimer level and ESR. Cerebrospinal fluid (CSF) analysis showed the following: pressure 210 mmH_2_O, leukocyte count 250*10^6^/L (72% polymorphonuclear, 28% monocytes) without altered erythrocytes, protein 1.10 g/l and glucose 2.99 mmol/l, and the CSF was absent for oligoclonal bands. Cytological examination of the CSF showed no malignant cells. CSF cultures were negative for bacteria, fungi, and mycobacteria, and polymerase chain reaction to test for herpes simplex virus (HSV PCR) was negative.

The patient was treated empirically with intravenous levofloxacin and piperacillin-tazobactam for 7 days before being referred, but no significant efficacy was seen. Based on the cerebrospinal fluid analysis and the lack of treatment efficacy, the diagnosis of intracranial abscess was considered, and anti-infective treatment with vancomycin, meropenem, and ganciclovir was given for 5 days. The patient’s symptoms did not improve with these treatments. We performed next-generation sequencing, which showed positive results, mainly *Prevotella oris*, with 1328 sequences detected and a relative abundance of 49. 94%, and a small number of *Peptostreptococcus stomatis*, with 290 sequences detected and a relative abundance of 11. 13%. When the results of this testing became available, the vancomycin treatment was discontinued, and the patient was maintained on ceftriaxone and metronidazole.

**Fig. 2:**
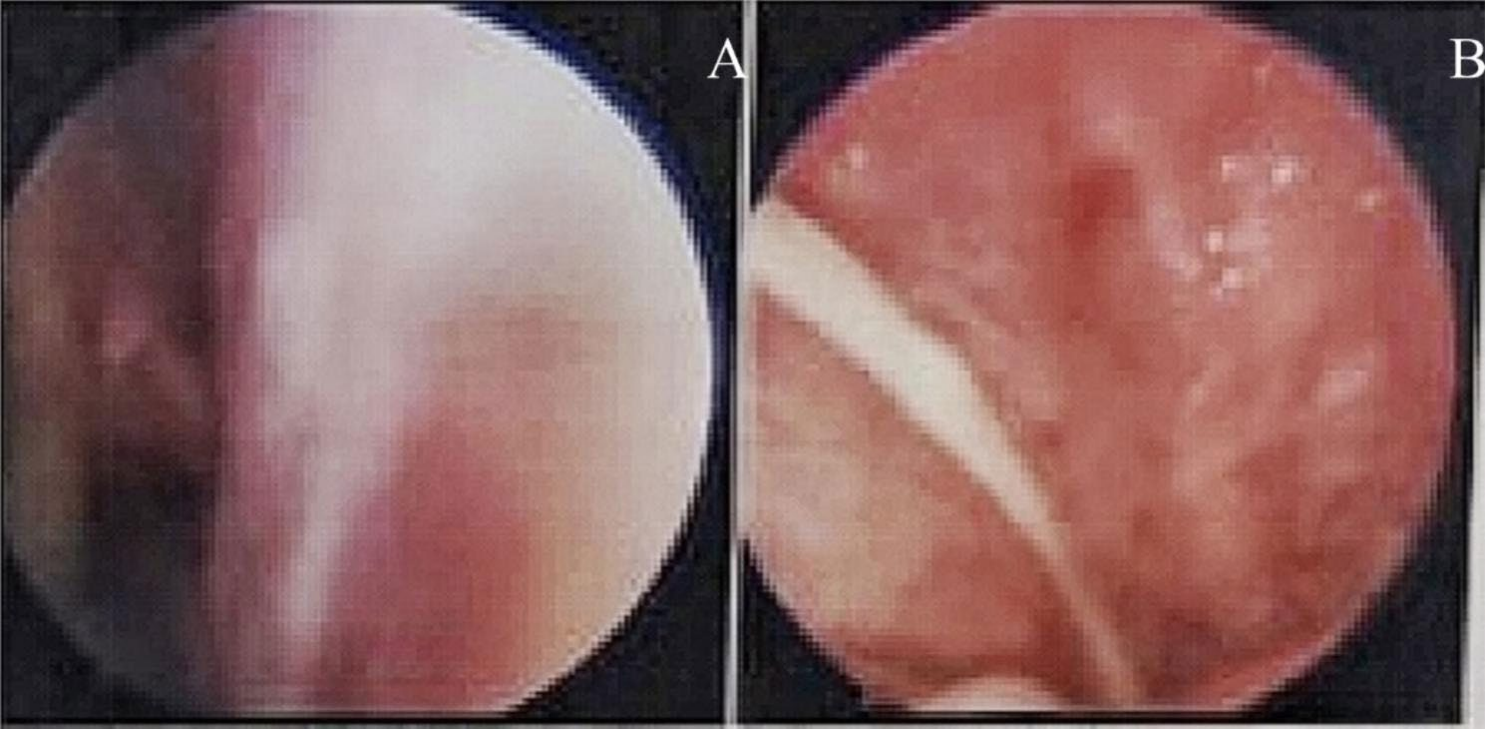
Nasal endoscopy showing an outflow of pus from the right sphenoid sinus ostium

Further investigations were performed to determine the source of the infection. We noted that brain MRI suggested inflammatory signals in the right septal and pterygoid sinuses. Nasal endoscopy revealed a purulent discharge visible in the right pterygoid sinus orifice (Fig. 2A-B), which also revealed *Prevotella oris*. Based on this finding, the revealed anaerobic bacteria could be logically responsible for a contiguous spread of infection following nasosinusitis.

**Fig. 3:**
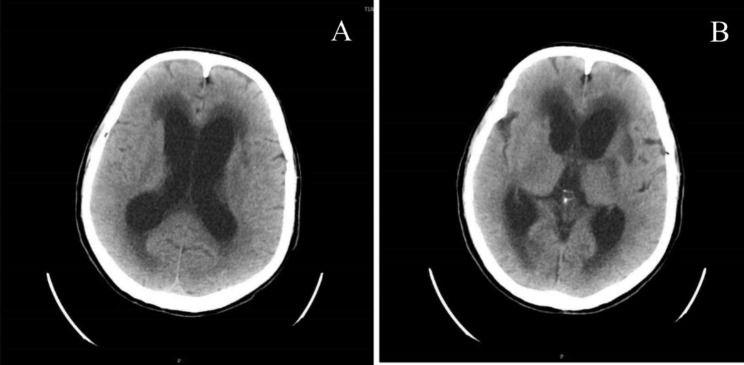
CT of the brain: dilatation of the supratentorial ventricular system and widening of the sulcioencephalic fissure

After 14 days of treatment, the patient’s symptoms were significantly improved, his consciousness was clear, and he could communicate normally. However, shortly after the symptoms improved, his condition worsened again, and he fell into a shallow coma and had speechlessness. Brain CT showed dilatation of the supratentorial ventricular system and widening of the sulcioencephalic fissure (Fig. 3A-B). We thought this was caused by hydrocephalus complicated by an intracranial abscess, so we transferred the patient to the neurosurgery department for ventriculoperitoneal shunting. After the operation, ceftriaxone and metronidazole were continued for 4 weeks, and the patient gradually recovered to his premorbid state and was soon discharged from the hospital. During the later follow-up process, we learned that he no longer had these symptoms.

## Discussion

A brain abscess is a focal suppurative process in the brain parenchyma, which can be a complication from paranasal sinusitis, otitis media, cranial trauma, or bacteremia from an extracranial focus of infection. Although the death rate from brain abscesses has declined from 40% in the 1950s to 10% in the early 21st century, it remains a fatal disease if untreated or without timely intervention. Timely identification of pathogenic bacteria and targeted anti-infection treatment can greatly improve the prognosis of patients.

In past cases, most of the patients had positive cultures for pathogenic bacteria on samples from pus drained from brain abscesses or from the blood. In this case, due to the complex form of intracranial infection, the specimen could not be obtained directly by surgical means. We were able to detect pathogenic anaerobic bacteria in the cerebrospinal fluid by next-generation sequencing, which has been successfully applied to the detection of pathogens and has greatly improved the accuracy of treatment and thus the prognosis of patients [3].

*Prevotella* spp. is a common group of obligate anaerobic bacteria in healthy humans. It mainly exists on the surface of the mucosa and has been isolated from the oral cavity and gastrointestinal and urogenital tract mucosa [4]. In 1982, Holdeman et al. first isolated a pathogenic bacterium from the oral cavity of a patient with periodontitis, attributed it to the genus *Bacteroides* and named it *Bacteroides oralis* [5]. In 1990, this bacterium was reclassified and named *Prevotella oris* by Shah et al. [6]. Some of the possible virulence factors for *Prevotella* spp. especially *P. pastoris*, include its unique 16 kDa hemolysin, ability to form a biofilm, and high fibronectin- and laminin-binding rates, which contribute to its invasiveness [7]. Cases of intracranial infection caused by *Prevotella oris* are very rare, and we searched PubMed for English language articles using the terms “*Prevotella oris* and brain abscess” and “brain abscess and *Bacteroides oris/oralis*”. A total of 6 articles with detailed case-specific information were identified, and they included 7 cases and are described in Table 1[2, 8–12].

**Table 1:** Reviewing the literature on brain abscess caused by *Prevotella oris*

References	No.	Age	Sex	Lesion location	Infectious source	Bacteria	Measurement technique	Clinical features	Management	Outcome
HR et al. [8]	Patient 1	16	Male	Cerebellum	Otogenic	*Bacteroides oralis* *Bacteroides fragilis* *Streptoccocci*	bacterial culture	No mention	excision metronidazole	Recovery
DN et al. [9]	Patient 2	21	Female	Frontal	Odontogenic	*Bacteroides oralis*	bacterial culture	Headache fever vomiting restlessness	penicillin metronidazole	Recovery
M et al. [10]	Patient 3	55	Female	Cerebellum	Otogenic	*Prevotella buccae* *Prevotella oris* *Bilophila wadsworthia* *Bacteroides fragilis Peptostreptococcus anaerobius*	bacterial culture	Headache dizziness vomiting	craniotomy penicillin metronidazole	Recovery
Shimumora et al. [11]	Patient 4	64	Female	Frontal	Meningioma	*Bacteroides oralis*	bacterial culture	Fever	craniotomy piperacillin gentamicin	Recovery
Cobo et al. [2]	Patient 5	24	Male	Frontal	Sinusitis	*Fusobacterium. necrophorum* *Prevotella oris* *Prevotella micra*	bacterial culture	Headache vomiting	excision cefotaxime metronidazole	Recovery
Cobo et al. [2]	Patient 6	18	Male	Frontal	Sinusitis	*Streptococcus. constellatus* *Prevotella oris*	bacterial culture	Headache fever vomiting	excision metronidazole linezolid	Recovery
Chen et al. [12]	Patient 7	20	Male	Frontal and fronto-parieto-temporal lobe	Odontogenic	*Porphyromonas endodontalis* *Prevotella oris* *Prevotella baroniae* *Fusobacterium nucleatum*	mNGS	Headache fever vomiting unconsciousness	excision meropenem ceftriaxone ornidazole linezolid	Recovery
our case	Patient 8	63	Male	Sellar and suprasellar regions	Sinusitis	*Prevotella oris* *Peptostreptococcus stoats*	mNGS	Headache fever intermittent diplopia	metronidazole ceftriaxone	Recovery

In previous case reports, most of the cases were brain abscesses that were confirmed by brain CT. In addition to brain abscesses in the sellar and suprasellar regions, our patient also showed signs of meningoencephalitis on brain MRI. This is what makes this case different from previous cases. In all cases, at least one of the symptoms of the classic triad (fever, headache, neurological deficits) was present, and headache was the most common. The main route of infection was due to the spread of sinusitis, followed by otitis media and periodontitis. In terms of treatment, most patients received neurosurgical treatment, including craniotomy and puncture drainage, and a few patients received only medical treatment, such as this patient. However, all patients had a good prognosis, and no deaths occurred. All patients were treated with metronidazole, which was effective, and treatment with metronidazole conformed to the recommended treatment for brain abscess [13].

## Conclusion

Intracranial infections caused by *Prevotella oris* are very rare in immunocompetent patients and are usually caused by the spread of inflammation in adjacent organs. We report a case of an intracranial infection caused by sinusitis in an elderly patient, and the prognosis was good after treatment with metronidazole and ceftriaxone. It is suggested that when patients present with intracranial infection complicated with sinusitis, otitis media, and periodontitis, attending physicians should consider the possibility of *Prevotella oris* infection, improve relevant examinations early, and give symptomatic treatment as soon as possible to effectively improve the quality of life of patients.

## Data Availability

All data generated or analyzed during this study are included in this published article. The datasets generated and analysed during the current study are available in the “Genome Sequence Archive” repository, persistent “CRA010431” to datasets.

## Abbreviations

MRI: Magnetic resonance imaging
mNGS: Metagenomic next-generation sequencing
ESR: Erythrocyte sedimentation rate
CSF: Cerebrospinal fluid
HSV PCR: Polymerase chain reaction to test for herpes simplex virus
CT: Computerized tomography

## References

[1] Vishwanath S, Shenoy PA, Gupta A (2016). Brain abscess with anaerobic gram-negative bacilli: Case series [J]. J Case Rep.

[2] Cobo F, Martin-Hita L, Navarro-Marí JM. Brain abscesses caused by anaerobic bacteria [J]. Anaerobe, 2022, 76: 102614,10.1016/j.anaerobe.2022.102614. 10.1016/j.anaerobe.2022.102614 35843460

[3] Stebner A, Ensser A, Geißdörfer W (2021). Molecular diagnosis of polymicrobial brain abscesses with 16s-rdna-based next-generation sequencing. Clin Microbiol Infect.

[4] Sharma G, Garg N, Hasan S et al. Prevotella: An insight into its characteristics and associated virulence factors [J]. Microb Pathog, 2022, 169: 105673,10.1016/j.micpath.2022.105673. 10.1016/j.micpath.2022.105673 35843443

[5] Holdeman LV, Moore WEC, Churn PJ (1982). Bacteroides oris and bacteroides buccae new species from human periodontitis and other human infections [J]. Int J Syst Evol MicroBiol.

[6] Shah HN, Collins DM (1990). Prevotella, a new genus to include bacteroides melaninogenicus and related species formerly classified in the genus bacteroides. Int J Syst Bacteriol.

[7] Viswanath LS, Gunalan A, Jamir I et al. Prevotella oris: A lesser known etiological agent of pleural effusion [J]. Anaerobe, 2022, 78: 102644,10.1016/j.anaerobe.2022.102644. 10.1016/j.anaerobe.2022.102644 36116686

[8] Ingham HR, Slekon JB, Roxby CM. Bacteriological study of otogenic cerebral abscesses: Chemotherapeutic role of metronidazole [J]. Br Med J, 1977, 2(6093): 991–993,10.1136/bmj.2.6093.991. 10.1136/bmj.2.6093.991 PMC1631786 922400

[9] Fredericka DN. Endocarditis and brain abscess due to bacteroides oralis [J]. J Infect Dis, 1982, 145(6): 918,10.1093/infdis/145.6.918. 10.1093/infdis/145.6.918 7086201

[10] Marina M, Ivanova K, Ficheva M et al. Bilophila wadsworthiain brain abscess: Case report [J]. Anaerobe, 1997, 3(2–3): 107–109,10.1006/anae.1997.0084. 10.1006/anae.1997.0084 16887572

[11] Shimomura T, Hori S, Kasai N et al. Meningioma associated with intratumoral abscess formation–case report [J]. Neurol Med Chir (Tokyo), 1994, 34(7): 440–443,10.2176/nmc.34.440. 10.2176/nmc.34.440 7526233

[12] Chen M, Lai Z, Cheng M et al. Rare brain and pulmonary abscesses caused by oral pathogens started with acute gastroenteritis diagnosed by metagenome next-generation sequencing: A case report and literature review [J]. Front Cell Infect Microbiol, 2022, 12: 949840,10.3389/fcimb.2022.949840. 10.3389/fcimb.2022.949840 PMC9561126 36250052

[13] Boyanova L, Kolarov R, Gergova G et al. Trends in antibiotic resistance in prevotella species from patients of the university hospital of maxillofacial surgery, sofia, bulgaria, in 2003–2009 [J]. Anaerobe, 2010, 16(5): 489–492,10.1016/j.anaerobe.2010.07.004. 10.1016/j.anaerobe.2010.07.004 20670687

